# Fractures Associated with Immune Checkpoint Inhibitors: A Disproportionality Analysis of the World Health Organization Pharmacovigilance Database

**DOI:** 10.3390/ph18030333

**Published:** 2025-02-26

**Authors:** Takenao Koseki, Hirofumi Hamano, Masakazu Hatano, Takao Tobe, Ryo Ieda, Tsuyoshi Nakai, Yoshito Zamami, Shigeki Yamada

**Affiliations:** 1 Department of Pharmacotherapeutics and Informatics, Fujita Health University School of Medicine, Toyoake 470-1192, Japan; hatanomasakazu@yahoo.co.jp (M.H.); takao.tobe@fujita-hu.ac.jp (T.T.); tsuyoshi.nakai@fujita-hu.ac.jp (T.N.); syamada@fujita-hu.ac.jp (S.Y.); 2 Department of Pharmacy, Okayama University Hospital, Okayama 700-8558, Japan; hamano.hirofumi@okayama-u.ac.jp (H.H.); zamami-y@okayama-u.ac.jp (Y.Z.); 3 Department of Clinical Pharmacology and Pharmacy, Okayama University, Okayama 700-8558, Japan

**Keywords:** disproportionality analysis, fractures, immune checkpoint inhibitors, hyperthyroidism, osteoporosis, pharmacovigilance, VigiBase

## Abstract

**Background/Objectives**: The risk of fractures associated with immune checkpoint inhibitors (ICIs) is increasing; however, the relationship between fracture risk and potential factors, such as osteoporosis and hyperthyroidism, remains unclear. **Methods**: Using VigiBase, the World Health Organization’s global pharmacovigilance database, we investigated the signals for osteoporosis, hyperthyroidism, and fractures associated with ICIs (nivolumab, pembrolizumab, atezolizumab, durvalumab, ipilimumab, and tremelimumab) by calculating information components (ICs) and their 95% confidence intervals (CIs). Furthermore, we estimated the association between the occurrence of fractures in patients receiving ICIs and osteoporosis or hyperthyroidism. **Results**: Signals of hyperthyroidism (IC = 4.66, 95% CI: 4.58–4.73), but not osteoporosis (IC = −1.79, 95% CI: −2.22 to −1.36) or fractures (IC = −0.21, 95% CI: −0.36 to −0.06), were detected in patients using ICIs. Osteoporosis (odds ratio: 118.00, 95% CI: 61.00–230.00) was associated with an increased reporting frequency of fractures related to ICIs, whereas hyperthyroidism (odds ratio: 0.60, 95% CI: 0.19–1.87) was not associated with such an increase. **Conclusions**: The VigiBase analysis indicates that the use of ICIs does not increase the reporting frequency of osteoporosis or fractures. Additionally, hyperthyroidism did not increase the reporting frequency of fractures associated with ICIs.

## 1. Introduction

Advances in cancer immunotherapy using immune checkpoint inhibitors (ICIs) have resulted in a paradigm shift in cancer treatment. ICIs inhibit immune checkpoint proteins, such as programmed cell death protein 1 (PD-1), programmed death-ligand 1 (PD-L1), and cytotoxic T-lymphocyte-associated antigen 4 (CTLA-4), thereby activating immunity through T lymphocyte activation and exerting anticancer effects. By contrast, ICIs can induce immune-related adverse events (irAEs) that are specific to these therapies. IrAEs are off-target toxic effects resulting from an excessively activated immune system. IrAEs can occur in any organ system, including the respiratory, cardiovascular, central nervous, gastrointestinal, hematological, and musculoskeletal systems, and skin [1].

A recent report proposed that osteoporotic fractures may be an unrecognized adverse event [2]. It suggests that T cells activated by ICIs may increase the expression of the receptor activator of nuclear factor-κB ligand (RANKL) and other cytokines, including tumor necrosis factor (TNF)-α and interleukin (IL)-18, IL-17, and IL-12, potentially leading to the development of osteoporosis. Indeed, the activation of T cells is known to induce the production of RANKL and TNF-α [3,4,5]. Immune cells have been suggested to play a crucial role in bone remodeling [6,7]. Bone-marrow-derived macrophages are known to polarize into M1 macrophages under sustained RANKL stimulation, subsequently differentiating into osteoclasts [8]. Macrophages also enhance the release of TNF-α, IL-6, IL-1, IL-12, IL-18, IL-23, and reactive oxygen species [9,10]. In addition, myeloid-derived suppressor cells have been shown to differentiate into osteoclasts in the presence of RANKL and macrophage colony stimulating factor [11,12,13]. Thus, the enhancement of immune responses by ICIs and associated effects on bone remodeling may increase the risk of fractures; however, clinical evidence remains limited.

A case series in humans reported six patients treated with ICIs who exhibited fractures or resorptive bone lesions [14]. Vertebral compression fractures and resorptive lesions—including those of the shoulder, hand, and clavicle—were observed. Elevated bone resorption markers were noted in five of the six patients. Another case series reported that three patients treated with ICIs presented with vertebral fractures; one patient exhibited a calcaneal fracture [15]. In a retrospective administrative database study, the incidence rate ratio of sustaining a major fracture in the year after versus prior to ICI initiation was reported to be 2.43 (95% confidence interval [CI]: 1.34–4.27) among patients treated using ICIs [16]. Based on these reports, ICIs may promote osteoporosis and increase the risk of fractures.

In contrast, ICIs can also cause endocrinopathies, such as hyperthyroidism and hypothyroidism, as one of the irAEs [17,18,19]. Among them, hyperthyroidism has been associated with an increased risk of osteoporosis and fractures [20,21]. It is a representative endocrine abnormality caused by ICIs, with a relatively high incidence of 3.9–8.5% and 10.2–16.4% in patients receiving PD-1/PD-L1 and combination therapy with CTLA-4 inhibitors, respectively [22,23]. Clarifying the relationships between ICI fracture risk, osteoporosis, and hyperthyroidism will provide important evidence that will contribute to the proper use of ICIs.

Recently, pharmacovigilance signal detection studies have increasingly utilized large-scale databases of adverse drug reactions (ADRs) collected through spontaneous reporting systems [24,25]. Spontaneous reporting systems and pharmacovigilance databases play a crucial role in identifying previously unrecognized AEs that were not detected in clinical trials and in evaluating drug safety based on real-world use in specific populations and clinical settings [26,27]. In pharmacovigilance studies, signal detection methods such as disproportionality analysis using reporting odds ratios and information components (ICs) are employed to determine the incidence of AEs [28,29,30]. VigiBase, provided by the World Health Organization (WHO), is a global pharmacovigilance database containing spontaneous ADR reports. Since its inception in 1964, VigiBase has accumulated data on approximately 36.6 million patients and 92.6 million ADRs. This extensive database is particularly valuable for identifying signals of rare ADRs, such as fractures in patients treated with ICIs.

Therefore, this study analyzed VigiBase to investigate the following: first, the presence of signals for osteoporosis and hyperthyroidism, which are risk factors for ICI-associated fractures; second, the presence of signals for fractures; and third, the association between fractures and osteoporosis or hyperthyroidism in patients using ICIs.

## 2. Results

### 2.1. Signals of Osteoporosis and Hyperthyroidism

The ICs and their 95% CIs were calculated using a three-by-three contingency table (Table 1). The ICs for osteoporosis and hyperthyroidism, reported as the ADRs of ICIs, are shown in Table 2. Signals were not detected for osteoporosis for all ICIs (patients reported to use at least one ICI, IC [95% CI]: −1.79 [−2.22 to −1.36]) or each ICI (IC [95% CI]: nivolumab, −1.46 [−2.04 to −0.89]; pembrolizumab, −1.87 [−2.61 to −1.12]; atezolizumab, −2.23 [−3.67 to −0.79]; durvalumab, −1.92 [−3.96 to 0.12]; ipilimumab, −2.07 [−3.16 to −0.98]; tremelimumab, not applicable [N.A.]). When hyperthyroidism, a typical irAE of ICIs, was evaluated, signals were detected for all ICIs (IC [95% CI]: 4.66 [4.58–4.73]) and each ICI (IC [95% CI]: nivolumab, 4.87 [4.77–4.98]; pembrolizumab, 4.33 [4.18–4.47]; atezolizumab, 4.47 [4.25–6.49]; durvalumab, 4.65 [4.34–4.96]; ipilimumab, 4.89 [4.73–5.05]), excluding tremelimumab (IC [95% CI]: 1.25 [−0.42 to 2.92]).

### 2.2. Signals of Fractures

The IC values of the fractures, reported as ADRs of the ICIs, are shown in Table 3. Signals were not detected for fractures for all ICIs (IC [95% CI]: −0.21 [−0.36 to −0.06]) or each ICI (ICs [95% CIs]: nivolumab, −0.09 [−3.08 to 0.12]; pembrolizumab, −0.41 [−0.68 to −0.14]; atezolizumab, −0.03 [−0.37 to 0.43]; durvalumab, −0.38 [−1.12 to 0.37]; ipilimumab, −0.02 [−0.34 to 0.31]; tremelimumab, −0.55 [−2.59 to 1.50]).

### 2.3. Association Between Fractures and Osteoporosis or Hyperthyroidism in Patients with ICIs

To investigate the association between fractures and osteoporosis or hyperthyroidism in patients treated with ICIs, multivariable logistic regression analysis was performed. Covariates included age (≥65 years) and gender (female), which generally affect the occurrence of fractures. Multivariable analysis revealed that an age ≥ 65 years, female sex, and osteoporosis (but not hyperthyroidism) were associated with an increased reporting frequency of fractures in patients treated using ICIs (odds ratio [95% CI]: age ≥ 65 years, 1.67 [1.35–2.07]; female sex, 1.56 [1.27–1.91]; osteoporosis, 118.00 [61.00–230.00]; hyperthyroidism, 0.60 [0.19–1.87]) (Table 4).

## 3. Discussion

The acceleration of osteoporosis and the associated risk of fractures by ICIs has been proposed; yet, unexpectedly, our study did not detect any signals for either osteoporosis or fractures associated with ICI use using VigiBase. Previous reports have evaluated the fracture signals associated with ICIs in the FDA Adverse Event Reporting System (FAERS), a worldwide pharmacovigilance database with a focus on the U.S. [15,31]. Filippini et al. [15] reported that ICIs—classified as PD-1 (nivolumab, pembrolizumab, cemiplimab), PD-L1 (atezolizumab, durvarmab, avelumab), and CTLA-4 (ipilimumab, tremelimumab) inhibitors—showed several types of fracture signals among the preferred terms (PTs) defined by the Medical Dictionary for Regulatory Activities (MedDRA) high-level group term (HLGT) “Bone and Joint Injuries”. This included pathological fracture, spinal compression fractures, and femoral neck fractures. Liu et al. [31] reported that no ICIs (nivolumab, pembrolizumab, atezolizumab, durvalumab, or ipilimumab) were associated with signals of overall fracture; however, signals for certain PTs of fractures, such as spinal compression fracture, thoracic vertebral fracture, and osteoporotic fracture, were detected for several ICIs. This report also indicated that no osteoporotic signals were detected in each ICI. Based on these reports, ICIs may partially increase the risk of certain fractures (PT levels); however, they are unlikely to significantly increase the overall risk of fracture. Our results also support this finding, as no signals were detected in the 78 fracture PT groups encompassed under the HLGT “Fractures”. Given that ICIs activate T cells, which, in turn, stimulate osteoclasts by increasing RANKL and cytokine levels, the risk of osteoporosis and fractures associated with ICIs is considered to occur uniformly, regardless of the target of action of the ICIs (i.e., PD-1, PD-L1, or CTLA-4). In the aforementioned studies, fracture and osteoporosis signals were evaluated for each ICI, or for groups of ICI targets of action. However, in this study, no fractures or osteoporosis signals were detected using VigiBase, even when all six ICIs were evaluated as a group. Therefore, further evidence is needed, and more cautious discussions are required regarding the potential increased risk of osteoporosis and fractures associated with ICIs.

Additionally, fracture risk may be affected by both osteoporosis and hyperthyroidism. Although we detected a signal for hyperthyroidism associated with ICIs, hyperthyroidism did not affect the reporting frequency of ICI-related fractures. Based on our analysis using VigiBase, it is unlikely that hyperthyroidism (an irAE) increases the risk of ICI-associated fractures. A meta-analysis reported that hyperthyroidism (thyroid-stimulating hormone [TSH] < 0.45 mIU/L) increases the risk of fractures, with an even higher risk observed when TSH levels are <0.10 mIU/L [32]. One proposed mechanism is the expression of TSH receptors in osteoblasts and osteoclasts, where changes in TSH activity may accelerate bone turnover, thereby increasing fracture risk [33]. In contrast, a recent study investigating the association between thyroid dysfunction and osteoporosis using publicly available databases reported that hyperthyroidism, FT3, FT4, and TSH levels were not risk factors for osteoporosis [34]. Based on these findings, the fracture risk in hyperthyroidism may vary depending on factors such as the underlying mechanism, severity, duration of the condition, and TSH levels. In our study, hyperthyroidism did not affect the reporting frequency of ICI-associated fractures, suggesting that the pathological changes in hyperthyroidism caused by ICIs do not lead to the functional alterations that induce fractures. Factors associated with fractures induced by ICI were elderly age, female sex, and osteoporosis, which are commonly known to affect fractures. Furthermore, while the risk of developing osteoporosis due to ICIs does not appear to be elevated, patients at risk of osteoporosis should be closely monitored for potential fractures.

This study has some limitations; first, it should be noted that VigiBase—comprising individual case safety reports corrected from spontaneous reporting systems—is a passive reporting database with many biases, including under-reporting, over-reporting, and confounding by comorbidities. Second, the diagnosis of osteoporosis requires the measurement of bone turnover markers, such as bone-specific alkaline phosphatase and bone mineral density [35,36]. However, not all patients receiving ICIs undergo regular bone density measurements, raising the possibility of osteoporosis under-reporting. Third, the influence of risk factors for fractures, such as glucocorticoid therapy associated with ICI therapy, bone metastasis, and fall risk, was not excluded. Nevertheless, this study suggests results that differ from the newly proposed hypothesis of increased osteoporosis and fracture risk associated with ICIs; thus, the hypothesis presented is crucial and warrants further investigation.

## 4. Materials and Methods

### 4.1. Data Source

The WHO VigiBase database is one of the most extensive global pharmacovigilance databases with over 36 million reports collected from >130 countries worldwide, based on post-marketing pharmacovigilance activity. In this study, data from December 1964–2023 were downloaded and processed.

### 4.2. Dataset Creation

**Figure 1:**
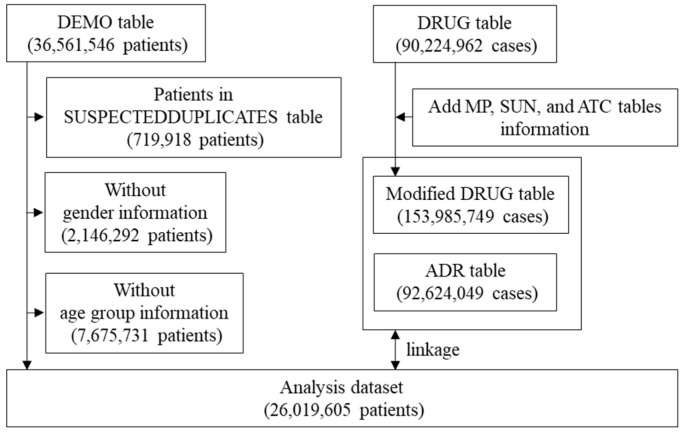
Flow chart for the creation of the disproportionality analysis dataset.

A flowchart illustrating the creation of the disproportionality analysis dataset is shown in Figure 1. First, suspected duplicates included in the “SUSPECTEDDUPLICATES” table—made by statistical algorithms developed by the World Health Organization Collaborating Centre—were excluded from the “DEMO” table. Additionally, after excluding patients of unknown sex and age, 26,019,605 patients were included in the analysis. Drug_name from the “MP” table, Substance_Id and Substance_name from the “SUN” table, and ATC_code from the “ATC” table were added to the “DRUG” table (Modified DRUG Table). The corresponding cases were extracted from the “Modified DRUG” and “ADR” tables according to the definitions of ICIs and ADRs, respectively, and linked to the analysis population to create an analysis dataset for the disproportionality analysis. VigiBase was processed using Navicat 16 in SQLite ver. 16.3.3 (PremiumSoft, Osaka, Japan).

### 4.3. Definition of Immune Checkpoint Inhibitors

Six ICIs (Substance Id) were included in the study: PD-1 inhibitors: nivolumab (27193) and pembrolizumab (28886); PD-L1 inhibitors: atezolizumab (32021) and durvalumab (31669); CTLA-4 inhibitors: ipilimumab (17640) and tremelimumab (18514, 38501). Nivolumab, pembrolizumab, atezolizumab, durvalumab, and ipilimumab were selected as representative ICIs, as they each had more than 3000 reported cases in the analysis dataset. Additionally, tremelimumab was included in the analysis since it is used in combination with durvalumab.

### 4.4. Definition of Adverse Drug Reactions

Osteoporosis, hyperthyroidism, and fractures were defined according to the PTs in MedDRA 26.0 J. Eleven PTs were associated with osteoporosis, as defined by Standardized MedDRA Queries (SMQs) for Osteoporosis/Osteopenia (SMQ code 20000178), and nineteen PTs were associated with hyperthyroidism, as defined by SMQs for Hyperthyroidism (SMQ code 20000161; Tables S1 and S2). Seventy-eight PTs were identified as fractures from PTs grouped under six high-level terms (HLTs) within the Fractures HLGT (HLGT code 10017322): seven PTs in Skull and Face Fractures (HLT code 10040958), four PTs in Thoracic Cage Fractures Non-spinal (HLT code 10072987), ten PTs in Spinal Column Fractures (HLT code 10072985), four PTs in Pelvic Fractures (HLT code 10034246), twenty-nine PTs in Limb Fractures (HLT code 10075885), and twenty-four PTs in Fractures NEC (HLT code 10072986; Table S3).

### 4.5. Statistical Analysis

In the disproportionality analysis, ICs were used to evaluate the signal detection of ADRs. The IC is an ADR signal index of the Bayesian Confidence Propagation Neural Network (BCPNN) analysis, calculated based on the Bayesian statistical approach developed by the WHO Uppsala Monitoring Center [29,37]. It can be used to detect ADR signals even in small samples [38,39]. The ICs and their 95% CIs were calculated using a three-by-three contingency table (Table 1) and equations as below. ADR signals were considered positive if the lower limit of the 95% CI for the IC was >0 [40].

IC equations:

$$\begin{array}{l} E\left({IC}_{11}\right)=\log_{2}\frac{\left(N_{11}+\gamma_{11}\right)\left(N_{++}+\alpha \right)\left(N_{++}+\beta \right)}{\left(N_{++}+\gamma \right)\left(N_{1+}+\alpha_{1}\right)\left(N_{+1}+\beta_{1}\right)}\\ V\left({IC}_{11}\right)={\left(\frac{1}{ln2}\right)}^{2}\left[\frac{N_{++}-N_{11}+\gamma -\gamma_{11}}{\left(N_{11}+\gamma_{11}\right)\left(1+N_{++}+\gamma \right)}+\frac{N_{++}-N_{1+}+\alpha -\alpha_{1}}{\left(N_{1+}+\alpha_{1}\right)\left(1+N_{++}+\alpha \right)}+\frac{N_{++}-N_{+1}+\beta -\beta_{1}}{\left(N_{+1}+\beta_{1}\right)\left(1+N_{++}+\beta \right)}\right]\\ \gamma =\gamma_{11}\frac{\left(N_{++}+\alpha \right)\left(N_{++}+\beta \right)}{\left(N_{1+}+\alpha_{1}\right)\left(N_{+1}+\beta_{1}\right)},\;\gamma_{11}=1,\;\alpha_{1}=\beta_{1}=1,\;\alpha =\beta =2\\ IC\left(95\%CI\right)=E\left({IC}_{11}\right)\pm 2\sqrt{V\left({IC}_{11}\right)} \end{array}$$

Multivariate logistic regression analyses were performed on data from patients using ICIs that were extracted from the analysis dataset. Statistical significance was set at *p* < 0.05. Statistical analyses were performed using EZR (Saitama Medical Center, Jichi Medical University, Saitama, Japan), a graphical user interface for R version 4.2.2 (The R Foundation for Statistical Computing, Vienna, Austria) based on a modified version of R Commander (version 1.61) designed to add statistical functions frequently used in biostatistics [41].

## 5. Conclusions

This study using VigiBase suggests that the ICI-associated osteoporosis incidence and consequent increase in fracture incidence are unlikely. Although ICIs may increase the incidence of hyperthyroidism, it is unlikely to lead to a higher incidence of fractures. Commonly known fracture risk factors such as elderly age, female sex, and osteoporosis also elevate the risk of fractures in patients using ICIs, emphasizing the need for caution irrespective of ICI use. Therefore, further investigation, including the use of other real-world databases, is needed to better understand the impact of ICIs on osteoporosis and fracture incidence.

## Figures and Tables

**Table 1:** Three-by-three contingency table.

	Target ADRs	Other ADRs	Total
Target ICIs	*N*_11_	*N*_10_	*N*_1+_
Other drugs	*N*_01_	*N*_00_	*N*_0+_
Total	*N*_+1_	*N*_+0_	*N*_++_

ADRs, adverse drug reactions, ICIs, immune checkpoint inhibitors.

**Table 2:** Information components of immune checkpoint inhibitors for osteoporosis and hyperthyroidism.

	*N*_11_	*N*_10_	*N*_01_	*N*_00_	IC [95% CIs]	Signal
Osteoporosis						
All ICIs	44	108,468	37,009	25,874,084	−1.79 [−2.22 to −1.36]	No
-Nivolumab	24	47,579	37,029	25,934,973	−1.46 [−2.04 to −0.89]	No
-Pembrolizumab	14	37,723	37,039	25,944,829	−1.87 [−2.61 to −1.12]	No
-Atezolizumab	3	12,472	37,050	25,970,080	−2.23 [−3.67 to −0.79]	No
-Durvalumab	1	4611	37,052	25,977,941	−1.92 [−3.96 to 0.12]	No
-Ipilimumab	6	19,912	37,047	25,962,640	−2.07 [−3.16 to −0.98]	No
-Tremelimumab	0	478	37,053	25,982,074	N.A.	N.A.
Hyperthyroidism						
All ICIs	1532	106,980	12,808	25,898,285	4.66 [4.58–4.73]	Yes
-Nivolumab	796	46,807	13,544	25,958,458	4.87 [4.77–4.98]	Yes
-Pembrolizumab	436	37,301	13,904	25,967,964	4.33 [4.18–4.47]	Yes
-Atezolizumab	174	12,301	14,166	25,992,964	4.47 [4.25–6.49]	Yes
-Durvalumab	88	4524	14,252	26,000,741	4.65 [4.34–4.96]	Yes
-Ipilimumab	354	19,564	13,986	25,985,701	4.89 [4.73–5.05]	Yes
-Tremelimumab	2	476	14,338	26,004,789	1.25 [−0.42 to 2.92]	No

*N*_11_, *N*_10_, *N*_01_, and *N*_00_ each represent the corresponding cell name in Table 1, which is represented by three-by-three contingency tables. CIs, confidence intervals; ICIs, immune checkpoint inhibitors; IC, information component; N.A., not applicable.

**Table 3:** Information components of immune checkpoint inhibitors for fractures.

	*N*_11_	*N*_10_	*N*_01_	*N*_00_	IC [95% CIs]	Signal
All ICIs	375	108,137	103,979	25,807,114	−0.21 [−0.36 to −0.06]	No
-Nivolumab	179	47,424	104,175	25,867,827	−0.09 [−3.08 to 0.12]	No
-Pembrolizumab	114	37,623	104,240	25,877,628	−0.41 [−0.68 to −0.14]	No
-Atezolizumab	51	12,424	104,303	25,902,827	−0.03 [−0.37 to 0.43]	No
-Durvalumab	14	4598	104,340	25,910,653	−0.38 [−1.12 to 0.37]	No
-Ipilimumab	79	19,839	104,275	25,895,412	−0.02 [−0.34 to 0.31]	No
-Tremelimumab	1	477	104,353	25,914,774	−0.55 [−2.59 to 1.50]	No

*N*_11_, *N*_10_, *N*_01_, and *N*_00_ each represent the corresponding cell name in Table 1, which is represented by three-by-three contingency tables. CIs, confidence intervals; ICIs, immune checkpoint inhibitors; IC, information components.

**Table 4:** Multivariable logistic analysis for the occurrence of fractures in patients with immune checkpoint inhibitors.

	Odds Ratio [95% CI]	*p*-Value
65 years or older (vs. under)	1.67 [1.35–2.07]	<0.001
Female (vs. male)	1.56 [1.27–1.91]	<0.001
Osteoporosis	118.00 [61.00–230.00]	<0.001
Hyperthyroidism	0.60 [0.19–1.87]	0.377

CI, confidence interval.

## Data Availability

Data are available upon reasonable request.

## Supplementary Materials

The following supporting information can be downloaded at: https://www.mdpi.com/article/10.3390/ph18030333/s1, Table S1: Definition of osteoporosis; Table S2: Definition of hyperthyroidism; Table S3: Definition of fractures.

## Abbreviations

The following abbreviations are used in this manuscript:

ADRs	Adverse drug reactions
ICIs	Immune checkpoint inhibitors
BCPNN	Bayesian Confidence Propagation Neural Network
WHO	World Health Organization
HLTs	High-level terms
SMQs	Standardized MedDRA Queries
TSH	Thyroid-stimulating hormone
MedDRA	Medical Dictionary for Regulatory Activities
HLGT	High-level group term
FAERS	FDA Adverse Event Reporting System
CIs	Confidence intervals
ICs	Information components
TNF-α	Tumor necrosis factor-alpha
IL	Interleukin
RANKL	Receptor activator of nuclear factor-κB ligand
irAEs	Immune-related adverse events
CTLA-4	Cytotoxic T-lymphocyte-associated antigen 4
PD-L1	Programmed death-ligand 1
PD-1	Programmed cell death protein 1
