# Supplementary material for: Fractures Associated with Immune Checkpoint Inhibitors: A Disproportionality Analysis of the World Health Organization Pharmacovigilance Database
Source: Pharmaceuticals (Basel). 2025 Feb 26;18(3):333. doi: 10.3390/ph18030333 (PMC11945245; doi:10.3390/ph18030333)
Supplement: Supplementary file 1 [file pharmaceuticals-18-00333-s001.zip › pharmaceuticals-3421200-supplementary.pdf]

Supplementary Materials

Fractures associated with immune checkpoint inhibitors: A disproportionality analysis of the World Health Organization pharmacovigilance database

Takenao Koseki[1*], Hirofumi Hamano[2,3], Masakazu Hatano[1], Takao Tobe[1], Ryo Ieda[1], Tsuyoshi Nakai[1], Yoshito Zamami[2,3], and Shigeki Yamada[1]

[1] Department of Pharmacotherapeutics and Informatics, Fujita Health University School of Medicine, Aichi, Japan
[2] Department of Pharmacy, Okayama University Hospital, Okayama, Japan
[3] Department of Clinical Pharmacology and Pharmacy, Okayama University, Okayama, Japan
*Correspondence: tkoseki@fujita-hu.ac.jp

Table of Contents

Table S1. Definition of osteoporosis
Table S2. Definition of hyperthyroidism
Table S3. Definition of fractures

Table S1. Definition of osteoporosis

SMQ code	SMQ name
20000178	Osteoporosis/osteopenia (SMQ)
**PT code**	**PT name**
10049470	Bone density decreased
10056809	Bone formation decreased
10065687	Bone loss
10064269	Bone marrow oedema syndrome
10088561	Idiopathic juvenile osteoporosis
10049088	Osteopenia
10031282	Osteoporosis
10031285	Osteoporosis postmenopausal
10031290	Osteoporotic fracture
10038642	Resorption bone increased
10039984	Senile osteoporosis

PT, Preferred term; SMQ, Standard MedDRA Queries.

Table S2. Definition of hyperthyroidism

SMQ code	SMQ name
20000161	Hyperthyroidism (SMQ)
**PT code**	**PT name**
10060742	Endocrine ophthalmopathy
10015683	Exophthalmos
10018706	Graves' disease
10067873	Hashitoxicosis
10020850	Hyperthyroidism
10083517	Immune-mediated hyperthyroidism
10075007	Inappropriate thyroid stimulating hormone secretion
10075386	Malignant exophthalmos
10068828	Marine Lenhart syndrome
10075899	Primary hyperthyroidism
10053260	Secondary hyperthyroidism
10069771	Thyroid dermatopathy
10043774	Thyroid tuberculosis
10075043	Thyrotoxic cardiomyopathy
10043786	Thyrotoxic crisis
10081524	Thyrotoxic myopathy
10043788	Thyrotoxic periodic paralysis
10075050	Toxic goitre
10044242	Toxic nodular goitre

PT, Preferred term; SMQ, Standard MedDRA Queries.

Table S3. Definition of fractures

HLGT code		HLGT name	
10017322		Fractures	
HLT code	HLT name	PT code	PT name
10040958	Skull and face fractures	10016042	Facial bones fracture
		10017310	Fractured skull depressed
		10040960	Skull fractured base
		10061365	Skull fracture
		10077603	Craniofacial fracture
		10085093	Craniofacial injury
		10085585	Osteo-meningeal breaches
10072987	Thoracic cage fractures non-spinal	10016747	Flail chest
		10039117	Rib fracture
		10042015	Sternal fracture
		10078358	Costal cartilage fracture
10072985	Spinal column fractures	10017308	Fractured sacrum
		10041541	Spinal compression fracture
		10041569	Spinal fracture
		10049164	Fractured coccyx
		10049946	Cervical vertebral fracture
		10049947	Lumbar vertebral fracture
		10049948	Thoracic vertebral fracture
		10052013	Spondylolysis
		10073162	Chance fracture
		10074807	Spinal fusion fracture
10034246	Pelvic fractures	10000397	Acetabulum fracture
		10021343	Ilium fracture
		10061161	Pelvic fracture
		10074362	Sacroiliac fracture
10075885	Limb fractures	10002544	Ankle fracture
		10009245	Clavicle fracture
		10016450	Femoral neck fracture
		10016454	Femur fracture
		10016667	Fibula fracture
		10016970	Foot fracture

Table S3. (*Continued*)

HLT code	HLT name	PT code	PT name
10075885	Limb fractures	10016997	Forearm fracture
		10017107	Fracture of clavicle due to birth trauma
		10018720	Greenstick fracture
		10019114	Hand fracture
		10020100	Hip fracture
		10020462	Humerus fracture
		10034122	Patella fracture
		10037802	Radius fracture
		10039579	Scapula fracture
		10043827	Tibia fracture
		10045375	Ulna fracture
		10048049	Wrist fracture
		10049128	Shoulder fracture
		10053962	Epiphyseal fracture
		10061394	Upper limb fracture
		10061599	Lower limb fracture
		10066094	Torus fracture
		10070073	Scapulothoracic dissociation
		10070884	Atypical femur fracture
		10073853	Osteochondral fracture
		10074551	Limb fracture
		10078749	Lisfranc fracture
		10081343	Maisonneuve fracture
10072986	Fractures NEC	10010149	Complicated fracture
		10010214	Compression fracture
		10017076	Fracture
		10023149	Jaw fracture
		10028200	Multiple fractures
		10030527	Open fracture
		10031290	Osteoporotic fracture
		10034156	Pathological fracture
		10042212	Stress fracture
		10049514	Traumatic fracture

Table S3. (*Continued*)

HLT code	HLT name	PT code	PT name
10072986	Fractures NEC	10052614	Comminuted fracture
		10053206	Fracture displacement
		10064210	Bone fissure
		10064211	Bone fragmentation
		10066184	Avulsion fracture
		10066386	Impacted fracture
		10069135	Periprosthetic fracture
		10072395	Atypical fracture
		10079667	Metaphyseal corner fracture
		10080404	Pseudofracture
		10080550	Osteophyte fracture
		10085543	Neurogenic fracture
		10087273	Depressed fracture
		10079864	Subchondral insufficiency fracture

HLT. High Level Group Term; HLGT, High Level Term; NEC, Not Elsewhere Classified; PT, Preferred term; SMQ, Standard MedDRA Queries.
